# Association Between New 340B Program Participation and Commercial Insurance Spending on Outpatient Biologic Oncology Drugs

**DOI:** 10.1001/jamahealthforum.2023.1485

**Published:** 2023-06-23

**Authors:** Jessica Chang, Pinar Karaca-Mandic, Sayeh Nikpay, Molly Moore Jeffery

**Affiliations:** 1 Division of Health Policy and Management, School of Public Health, University of Minnesota, Minneapolis; 2 Department of Finance, Carlson School of Management, University of Minnesota, Minneapolis; 3 Division of Health Care Delivery Research, Mayo Clinic, Rochester, Minnesota

## Abstract

**Importance:**

Previous studies have found that hospitals participating in the 340B Drug Pricing Program have higher Medicare Part B spending and expansion into affluent neighborhoods. Less is known about the association of 340B participation with spending by commercial insurance, where reimbursements are higher than Medicare.

**Objective:**

To use the Affordable Care Act expansion of eligibility for the 340B Drug Pricing Program to study the association between participation and spending on outpatient-administered oncological drugs for commercially insured patients.

**Design, Setting, and Participants:**

This cohort study included a balanced panel hospital cohort containing new and never 340B program participants between 2007 and 2019; more recent data were not included to avoid the effect of disruptions in care due to the COVID-19 pandemic. Descriptive analyses documented spending trends for patients receiving common outpatient-administered biologics used in cancer treatments (bevacizumab, filgrastim, pegfilgrastim, rituximab, and trastuzumab) at 340B (treated) and non-340B (control) hospitals. A difference-in-differences model assessed changes in episode drug spending. Analyses were conducted between December 2021 and June 2022.

**Exposure:**

New 340B program participation between 2010 and 2016.

**Main Outcome and Measures:**

Total drug episode spending, with control variables including total billed units, drug, calendar-year fixed effects, and hospital fixed effects.

**Results:**

Of 95 127 included episodes (56 917 [59.8%] episodes in female patients) across 478 hospitals, patients seen in 340B and non-340B hospitals were similar in sex and drug used, and 340B hospital patients were older than non-340B patients (median [IQR] age for all patients, 61 [51-71] years). New 340B participating hospitals were more likely to be small (<50 beds) and more likely to be in rural settings. In the difference-in-differences analysis, total episode drug spending increased by $4074.69 (95% CI, $1592.84-$6556.70; *P* = .001) in the year following start of 340B program participation relative to nonparticipants. Heterogeneous group time effects were seen, with earlier participants less likely to have increased episode spending.

**Conclusions and Relevance:**

In this cohort study, new 340B participation was associated with statistically significant higher oncological drug episode spending compared with nonparticipants after changes in 340B inclusion rules in 2010. These findings raise questions about unintended consequences of the 340B program on drug spending from the commercially insured population.

## Key Points

**Question:**

Is the 340B Drug Pricing Program associated with changes in outpatient biologic oncology drug spending among commercially insured patients?

**Findings:**

In this cohort study of 95 127 outpatient biologic oncology drug episodes in 478 hospitals, total episode spending increased $4074.69 in the first year of 340B participation relative to nonparticipating hospitals, with continued higher spending in the first 3 years of participation.

**Meaning:**

The increases in commercial payer spending for these drugs following 340B program participation may suggest an unintended program effect.

## Teaser

This cohort study uses the Affordable Care Act expansion of eligibility for the 340B Drug Pricing Program to study the association of program participation with spending on outpatient-administered oncological drugs for commercially insured patients.

## Introduction

Nonretail drugs—those administered in medical settings rather than used at home—accounted for about one-third of US prescription drug spending in 2018.^1^ Costs for these drugs are projected to increase faster than retail drugs.^2^ The majority of cancer drugs are delivered in health care settings.^3,4^ The burden of rising costs for drugs may directly affect patient health when patients cannot afford their medications. For example, Medicare enrollees who did not qualify for drug subsidies abandoned 30% of oncologic drug prescriptions.^5^

The 340B Drug Pricing Program was designed to allow safety-net hospitals to defray the rising cost of drugs through mandated discounts on outpatient drugs. To be eligible for the 340B program, hospitals had to meet the disproportionate share hospital (DSH) threshold (11.75% at time of publication).^6^

Recently, critics have questioned whether the 340B program has achieved its intended goal of expanding charity health care to vulnerable patient populations. Several studies showed that the 340B program was not associated with more uncompensated care or offering lower-profit hospital service lines^7^ and that 40% of 340B hospitals provided less than the national average share of uncompensated care.^8^

A growing literature suggests that 340B participation is associated with increases in hospital income both directly by lowering drug acquisition costs for program participants as well as through changes in delivery of care. Several studies have found that participation in the program was associated with hospital–clinic vertical integration (eg, hospital acquisition of hematology/oncology practices) and consolidation of outpatient drug administration into hospital-owned facilities.^9^ Participation is also associated with a shift in the site of cancer drug administration from physician offices to hospital outpatient departments among Medicare fee-for-service beneficiaries.^9,10^ Because drugs administered in hospital outpatient departments generate higher payments than those administered in physician offices,^11^ this shift in site of care is a potential source of increased revenue for 340B hospitals. Participation in 340B has also been shown to be associated with expansion of clinic sites into more affluent communities, which may also offer opportunities to increase revenue due to favorable payer mix.^12^ Expansion into affluent communities can also potentially increase the market share as well as the bargaining power of hospital systems with commercial insurers. Increases in both market share and bargaining power can lead to higher negotiated prices charged by hospitals for outpatient drugs.

The passage of the Affordable Care Act in 2010 expanded 340B eligibility to freestanding cancer hospitals and critical access hospitals regardless of their DSH status and lowered the DSH threshold for rural referral centers and sole community hospitals.^13^ Participants in the 340B drug program can purchase most medications at lower costs.^14^ A 2015 report by MedPAC found that 340B participants received discounts of 20% to 50% on most outpatient drugs.^15^ The program does not place restrictions on use of 340B-acquired drugs by patients’ insurer status: drugs purchased through the program can be administered to patients with commercial insurance.

To date, little is known about the association of 340B program participation with the total spending (health plan– plus patient-paid amounts) on outpatient drugs for patients with commercial insurance. Unlike traditional Medicare in which reimbursement amounts are set administratively, the reimbursement amounts paid by commercial insurers are negotiated between the insurers and hospitals. Because much of the prior literature has used data from Medicare, less is known about the influence of 340B participation on payments from commercial insurers and commercially insured patients. In this cohort study, we used the Affordable Care Act expansion of eligibility for the 340B program to study the association of program participation with spending on outpatient-administered oncological drugs for commercially insured patients.

## Methods

### Data Sources

We used administrative claims data from OptumLabs Data Warehouse between 2007 and 2019, which included deidentified pharmacy and medical claims and enrollment records for commercially insured enrollees from a single carrier across the US. More recent data were not included to avoid the effect of disruptions in care due to the COVID-19 pandemic. The OptumLabs Data Warehouse contains longitudinal health information on enrollees representing a diverse mixture of ages, races and ethnicities, and geographical regions across the US.^16^ The 340B program registry data were linked by OptumLabs using a combination of insurer identifiers such as National Provider Identifiers and/or Medicare Provider Numbers, and we received a deidentified and encrypted version of the linked claims data. Hospital characteristics (eg, bed size, rurality, ownership type, total outpatient volume) were linked from the American Hospital Association’s Annual Survey for the years 2014 and 2015. Analyses were conducted between December 2021 and June 2022.

Data were statistically deidentified and exempt from institutional review board review. This study followed the Strengthening the Reporting of Observational Studies in Epidemiology (STROBE) reporting guideline for cohort studies.

### Study Population

We restricted the study to facility claims for 5 outpatient biologic oncology drugs of interest (filgrastim, pegfilgrastim, trastuzumab, bevacizumab, and rituximab) using the Healthcare Common Procedure Coding System. To account for different billing patterns among hospitals, we constructed episodes of outpatient-administered drug use. We defined an episode as a period with 1 or more drug administrations and fewer than 31 days between filgrastim treatments, 42 days between pegfilgrastim treatments, 42 days between bevacizumab treatments, 56 days between rituximab treatments, or 30 days between trastuzumab treatments. These time frames were empirically selected by analyzing time between administrations of each drug type, with the cutoff set at the 90th percentile. We excluded episodes if drugs were administered at multiple hospitals during the episode (n = 1522 [1.6%]).

The unit of analysis was a patient-episode-year. We conducted descriptive statistical analyses on patients receiving treatments at study hospitals and characteristics of those hospitals to compare characteristics between the 340B and non-340B hospitals. We also examined patient characteristics such as age (categorical), sex, state of residence, insurance coverage (commercial or Medicare Advantage), and type of drug administered, as well as hospital characteristics such as bed size, rurality, and ownership.

We used claims from 2007 to 2019 to construct a balanced event study analytical data set of hospitals that were never 340B program participants and new 340B program participants (ie, in or after 2010). Hospitals were required to have at least 1 drug episode in the data set for at least 7 consecutive years for inclusion in the study sample. We excluded hospitals already participating in 340B prior to the study period. The treatment group included hospitals that initiated 340B program participation between 2010 and 2016. A set of control hospitals never participated in 340B; to maintain a balanced panel, a sham participation date was randomly assigned to nonparticipating hospitals and used to select their study inclusion period. It was not used in the event study analysis.

### Study Outcomes

The primary study outcome was total drug spending for included drugs in patient treatment episodes. Total spending was calculated as the allowed amount—that is, the sum of health plan–paid amounts and out-of-pocket amounts from claims. We included only spending attributed to the administered drugs (identified through Healthcare Common Procedure Coding System codes). Other services rendered during the course of care, including administration fees, laboratory testing, and other tests, were excluded from the total spending outcome. We did not observe whether balance billing occurred (ie, whether the hospital submitted a bill to the patient for the amount not covered by insurance), nor whether patients participated in pharmaceutical assistance programs. Administered drugs are typically billed by units administered to patients by the health care professional. However, because unit prices are not reported in commercial claims, and because billing patterns differ across providers (eg, physicians, hospitals, health care practices), we were not able to use unit price as the outcome variable.

All drugs included in the study were administered in the outpatient setting; use of drugs dispensed for self-administration/home use was excluded. The secondary study outcome was volume of drug administration visits at the hospitals during the study period. Drug administration visits are defined as distinct patient-drug-service date.

### Statistical Analysis

The empirical strategy examined changes in the outcome variable before and after new 340B-participating hospitals (treated hospitals) relative to hospitals that never participated in the 340B program (control hospitals). We used a time-varying difference-in-differences generalized linear regression model within an event-study framework to flexibly specify the treatment effect.^17^ In addition to a time-varying treatment indicator (differences-in-differences) or a set of indicators for periods before and after treatment (event study), the analyses included hospital-level and year-level indicators to complete the model. We also included indicators for drug type (bevacizumab, trastuzumab, filgrastim, pegfilgrastim, and rituximab) to account for level differences in reimbursement across drugs. The reference time period in both models was set to the first year of program participation. We selected the first year of participation because changes in negotiated prices would likely not occur until the second year of participation. We then ran the same regression specification separately for commercial and Medicare Advantage enrollees to account for different price setting between hospitals and payers between insurance segments. Specifically, Medicare Advantage reimbursement is generally lower than commercial insurance reimbursement for the same services and may follow Medicare fee-for-service reimbursement rates.^18,19^

The key exposure variables captured the length of time (3 years preparticipation and 3 years postparticipation) from the first year of 340B participation by the hospital and as such were a set of 6 indicator variables set to 1 if the patient episode was in a 340B-participating hospital at the given year relative to the first year of 340B participation (the treatment year). These 6 indicators were set to zero for patient-episodes in hospitals that never participated in 340 programs. The models also adjusted for the patient and hospital characteristics described previously.

For event study estimates to be unbiased estimates of the treatment effect, there are some key assumptions. We do not consider this analysis to yield causal estimates, but describing the assumptions may assist readers in assessing the meaning of the findings. First, there should be no co-occurring treatment; this is not verifiable empirically, but we are unaware of co-occurring shocks that could have lowered drug prices and changed treatment behavior. Second, the treatment and control groups should have similar trends before treatment that would continue in the absence of the intervention. We used an *F* test to assess the joint statistical significance of the 3 preperiod point estimates. Finally, expressing a single estimate of the treatment effect assumes the estimated treatment effects are constant across cohorts. To assess this assumption, we performed robustness checks estimating group-time effects and average treatment effects by length of treatment exposure, following the methods of Callaway and Sant’Anna.^20^ These analyses take different approaches to estimating the treatment effects and are further described in the eMethods in Supplement 1. Analyses were performed using Stata, version 16 (StataCorp), and *P* < .05 was considered statistically significant using 2-sided tests.

## Results

### Descriptive Statistics

The Table summarizes the descriptive characteristics of hospitals included in the analytical sample. A total of 305 hospitals that became 340B program participants between 2012 and 2017 (treated hospitals) were initially identified. Of these, 121 hospitals were excluded from the analytical sample due to balanced inclusion criteria (ie, they did not have ≥1 eligible episodes in ≥1 study years). The final analytical sample included 214 treated hospitals and 264 control hospitals. Treated 340B hospitals in the sample were smaller, with 17.4% of hospitals having bed sizes between 0 to 49 compared with just 4.2% of control hospitals, and treated hospitals were more likely in rural settings compared with non-340B control hospitals (15.8% vs 6.4%; *P* = .005) (Table). Both 340B and non-340B hospitals had similar outpatient visit volumes prior to 340B program participation (mean annual visits, 288 187.30 vs 332 290.60; *P* = .29).

**Table.** Descriptive Characteristics of Hospitals in the Study Sample

Variable	No. (%)		*P* value
	340B Hospitals (n = 214)	Non-340B hospitals (n = 264)	
No. of beds^a^			
0-49	31 (17.4)	9 (4.2)	<.001
50-199	56 (31.5)	79 (36.6)	
200-399	57 (32.0)	80 (37.0)	
≥400	34 (19.1)	48 (22.2)	
340B Start year			
2010	58 (22.0)	41 (19.2)	.07
2011	31 (11.7)	42 (19.6)	
2012	26 (9.9)	34 (15.9)	
2013	23 (8.7)	31 (14.5)	
2014	22 (8.3)	37 (17.3)	
2015	16 (6.1)	32 (15.0)	
2016	38 (14.4)	47 (22.0)	
Hospital type			
Rural^a^	26 (15.8)	12 (6.4)	<.001
Nongovernment^a^	176 (>95.0^b^)	177 (81.9)	<.001
Adjusted admissions, mean (SD)^a^	25 237.78 (27 716.18)	28 731.57 (20 304.92)	.15
Medicare discharges, mean (SD)^a^	4284.41 (4760.26)	6095.06 (4938.12)	.27
Medicaid discharges, mean (SD)^a^	1697.35 (1838.21)	1375.47 (1583.38)	.59
Total outpatient visits, mean (SD)^a^	288 187.30 (279 327.00)	332 290.60 (499 522.50)	.29

^a^ Hospital characteristics presented only for hospitals that completed the American Hospital Association survey.

^b^ Percentage displayed in adherence to data vendor’s cell-size suppression policy.

However, treated hospitals had more Medicaid discharges than control hospitals, as would be expected (mean annual discharges, 1697.35 vs 1375.47; *P* = .59). Of 95 127 outpatient biologic oncology drug episodes, 56 917 (59.8%) occurred in female patients, and the median (IQR) age for all patients was 61 (51-71) years. See eTable 2 in Supplement 1 for descriptive characteristics of patients who received oncologic outpatient treatment broken by 340B and non-340B hospitals.

**Figure 1.**
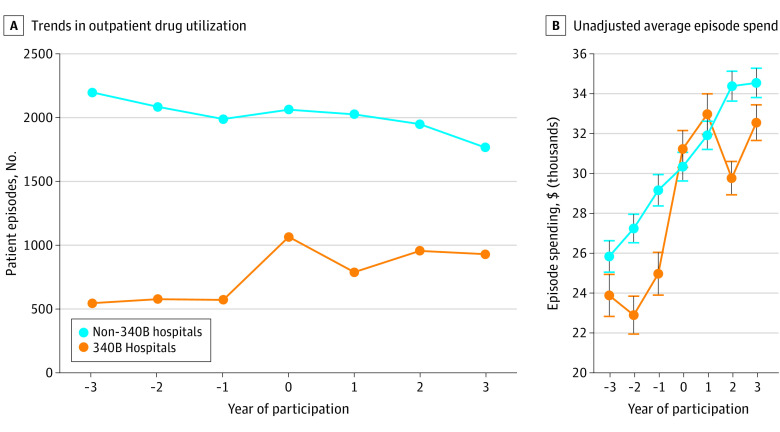
Trends in Outpatient Drug Utilization and Unadjusted Average Episode Spend Before and After 340B Drug Pricing Program Participation Utilization was defined as the number of patient episodes, and 0 represents the year of 340B program participation. For non-340B hospitals, a pseudo-340B start year was randomly assigned. Error bars indicate 95% CIs.

Figure 1A displays a descriptive event-study plot of average outpatient drug administration visits, which shows that treated hospitals increased observed outpatient drug visits by nearly 100% after 340B program participation (average of 500 outpatient visits preparticipation to 1000 outpatient visits postparticipation). Figure 1B displays the mean episode spending and 95% CIs between 340B and non-340B hospitals during the study period. Prior to 340B program participation, treated hospitals had lower average episode spending. For example, in the year prior to 340B program participation, average episode spending at treated hospitals was $25 009.53 (95% CI, $23 929.77-$26 089.29) compared with $29 227.12 (95% CI, $28 433.91-$30 200.33) at control hospitals. Episode spending at treated hospitals grew following 340B program participation. For example, in the year following 340B program participation, average episode spending at treated hospitals was $33 070.70 (95% CI, $32 044.35-$34 097.05) compared with $32 001.61 (95% CI, $31 288.98-$32 714.24) at control hospitals.

### Statistical Analysis

**Figure 2.**
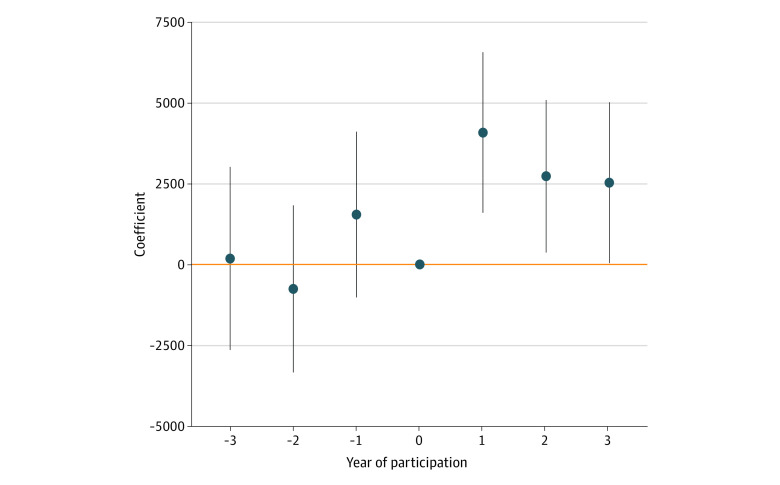
Event-Study Difference-in-Differences on Total Episode Spending Between 340B and Non-340B Hospitals, Pooled Sample Model included calendar year, event time, episode drug, hospital fixed effects, and patient characteristics (age group, insurance type, state of residence, and sex). Error bars indicate 95% CIs, and 0 represents the year of 340B Drug Pricing Program participation. Full model specification is included in the eMethods in Supplement 1.

**Figure 3.**
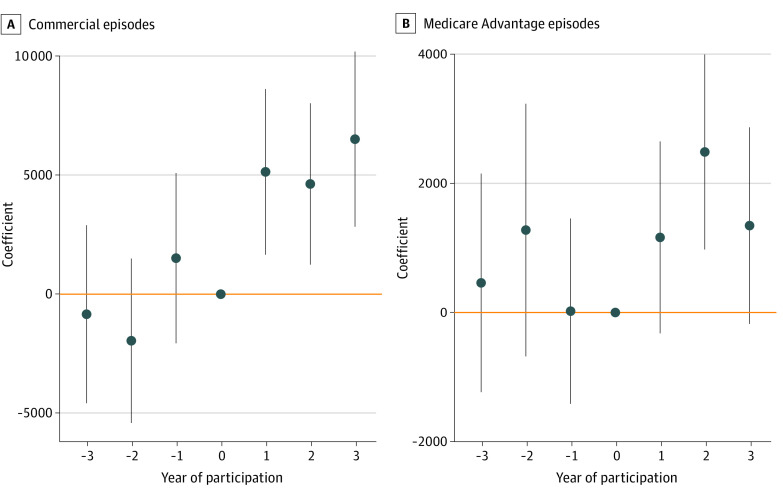
Event-Study Difference-in-Differences on Total Episode Spending Between 340B and Non-340B Hospitals by Insurer Segment Model included calendar year, event time, episode drug, hospital fixed effects, and patient characteristics (age group, state of residence, and sex). Error bars indicate 95% CIs, and 0 represents the year of 340B Drug Pricing Program participation. Full model specification is included in the eMethods in Supplement 1.

Figure 2 displays estimated event-study coefficients, which represent the adjusted changes between 340B and non-340B hospitals by time period among the pooled sample. See eTable 1 in Supplement 1 for complete results of the difference-in-differences regression model. There was no evidence of differential episode spending amount between 340B and non-340B hospitals 1, 2, and 3 years prior to 340 participation. Amounts paid for oncologic treatment episodes were statistically significantly higher for patients treated at 340B-participating hospitals compared with the amounts paid before 340B participation: year 1 of participation, $4074.77 (95% CI, $1592.84-$6556.70; *P* = .001); year 2 of participation, $2722.34 (95% CI, $364.68-$5080.00; *P* = .02); and year 3 of participation, $2521.17 (95% CI, $36.03-$5006.31; *P* = .047). Figure 3 displays the estimated event-study coefficients for commercial and Medicare Advantage episodes, respectively. There was no differential trend in episode spending amount between 340B and non-340B hospitals among patients with Medicare Advantage (Figure 3B): year 1 of participation, $1164.29 (95% CI, −$322.79 to $2649.37; *P* = .13); year 2 of participation, $2486.87 (95% CI, $976.21-$3997.54; *P* = .001); and year 3 of participation, $1346.02 (95% CI, −$176.34 to $2868.38; *P* = .08). On the other hand, there was a pronounced differential increase in episode spending amount between 340B and non-340B hospitals among commercial patients (Figure 3A): year 1 of participation, $5132.37 (95% CI, $1656.82-$8607.92; *P* = .004); year 2 of participation, $4625.08 (95% CI, $1239.56-$8010.60; *P* = .007); and year 3 of participation, $6504.18 (95% CI, $2829.79-$10 178.56; *P* = .001).

### Robustness Checks

A joint hypothesis test was conducted on the 3 pretreatment point estimates, which failed to reject the null hypothesis that the coefficients were jointly equal to zero, identifying no difference in pretrends between treated and control groups (*F* statistic, 0.88; *P* = .45). Following Callaway and Sant’Anna,^20^ average effects by length of exposure to treatment were assessed (eFigure 1 in Supplement 1); results were similar to the main analysis. An analysis of group-time treatment effects showed variation across the cohorts (eFigure 2 in Supplement 1). The cohorts initiating 340B participation in 2012, 2013, and 2015 had large, positive treatment effects. The cohorts initiating in 2010, 2011, 2014, and 2016 had treatment effects not statistically distinguishable from zero. No cohorts showed negative treatment effects.

## Discussion

We found that both spending for outpatient oncological biologic drug episodes and volume of episodes treated with these drugs increased after 340B program participation. The present analyses found that patients seen in hospitals newly participating in the 340B program spent more per drug episode. Consistent with existing literature on allowed amount correlation between Medicare fee-for-service and Medicare Advantage,^18,19^ we found that reimbursements from patients enrolled in Medicare Advantage were substantially lower than from patients enrolled in commercial health plans. When we stratified the analysis by patients with commercial insurance and patients with Medicare Advantage, we did not observe a differential increase in episode spending following 340B program participation among patients with Medicare Advantage. However, we did observe a sharper and more pronounced differential increase in episode spending following 340B program participation among patients with commercial insurance.

This analysis does not directly address the mechanism for the increased reimbursements after 340B participation. The episode definition used in this analysis means that there are a limited number of ways episode spending can change: (1) cost of the drug per dosing unit increases, (2) number of dosing units per visit increases, and (3) number of visits per episode increases. Dosing units per visit are relatively fixed, suggesting price increases per dosing unit or longer episodes of care are the most likely mechanisms. Prior studies have found that 340B participation is associated with a number of things that would tend to increase payer/patient-paid costs, including increased market share and bargaining power leading to increased prices and vertical integration allowing shifts in site of care from clinics to hospital facilities.^8,9^ The increased reimbursements we see could represent a direct increase in prices within a hospital or could represent a change in average price due to vertical integration of practices with higher prices. The present data are not able to distinguish between these cases.

### Limitations

Due to data limitations on the geographic detail and provider affiliations included in the data set, we were unable to assess whether the causal mechanism linking 340B participation and increases in outpatient drug spending was related to expanded market share and bargaining power, as has been hypothesized. This study is limited to claims and enrollment data from 1 national health plan, which limits generalizability of findings of outpatient drug spending to the broader commercial health plans. We were also not able to observe actual drug unit prices negotiated by health plans and hospitals, nor did we observe the actual 340B drug discounts provided to 340B hospitals by drug manufacturers. Nevertheless, the primary outcome of total episode drug spending served as an appropriate proxy outcome for unit price, as we would not expect hospitals to differentially increase intensity (ie, increase units of drugs administered) in treatment of patients with cancer following 340B program participation due to drug toxic effects and detrimental health adverse effects. We restricted the observations to hospitals with at least 1 outpatient drug claim in the data set (ie, with this carrier) for each of the drugs of interest 3 years preparticipation and postparticipation in the program. Therefore, this study may not be representative of hospitals that were excluded from health plan provider networks. Lastly, the estimates showed group time-specific effects that varied across cohorts.^20,21^ In particular, the cohorts initiating 340B participation in 2010, 2011, 2014, and 2016 did not show positive treatment effects. These cohorts (except for the 2016 cohort) tended to be smaller hospitals, with fewer admissions and outpatient visits (eTable 3 in Supplement 1). This may indicate that they had smaller market power compared with later 340B hospital participants, potentially limiting their ability to raise prices, but we are unable to definitively explain the differences across cohorts.

## Conclusions

This cohort study showed that in the years following 340B program participation, there were statistically significant increases in reimbursement for oncological drug episodes among treated hospitals. In the years prior to 340B program participation, we did not observe statistically significant episode reimbursement differences between the treated and control hospitals. The treated hospitals (new 340B program participants) were smaller in terms of bed sizes as well as located in rural communities compared with the control hospitals. The results from this study suggest that newly 340B-participating hospitals may benefit from increased market power, which they use to negotiate higher reimbursements for drug episodes among commercially insured patients. We hypothesize that hospital systems may be expanding services in off-campus offices following 340B program participation, although we were not able to observe these changes directly. Future studies on commercial prices and 340B program participation should incorporate 340B affiliated off-campus offices.

## Notes

Supplement 1. **eTable 1.** Difference-in-difference regression results **eTable 2.** Characteristics of patient episodes **eFigure 1.** Callaway-Sant’Anna average effect by length of exposure **eFigure 2.** Callaway-Sant’Anna group-time effects **eTable 3.** Additional descriptive analysis of hospital sample
eMethods

Supplement 2. Data Sharing Statement
