# Supplementary material for: Association Between New 340B Program Participation and Commercial Insurance Spending on Outpatient Biologic Oncology Drugs
Source: JAMA Health Forum. 2023 Jun 23;4(6):e231485. doi: 10.1001/jamahealthforum.2023.1485 (PMC10290244; doi:10.1001/jamahealthforum.2023.1485)
Supplement: Supplement 1. — eTable 1. Difference-in-difference regression results eTable 2. Characteristics of patient episodes eFigure 1. Callaway-Sant’Anna average effect by length of exposure eFigure 2. Callaway-Sant’Anna group-time effects eTable 3. Additional descriptive analysis of hospital sample eMethods [file jamahealthforum-e231485-s001.pdf]

Supplemental Online Content

Chang J, Karaca-Mandic P, Nikpay S, Jeffery MM. Association between new 340B program participation and commercial insurance spending on outpatient biologic oncology drugs. *JAMA Health Forum*. 2023;4(6):e231485. doi:10.1001/jamahealthforum.2023.1485

eTable 1. Difference-in-difference regression results
eTable 2. Characteristics of patient episodes
eFigure 1. Callaway-Sant'Anna average effect by length of exposure
eFigure 2. Callaway-Sant'Anna group-time effects
eTable 3. Additional descriptive analysis of hospital sample
eMethods

This supplemental material has been provided by the authors to give readers additional information about their work.

© 2023 Chang J et al. *JAMA Health Forum.*

eTable 1. Difference-in-difference regression results

**Outcome= Total outpatient drug spending**	**Coefficient**	**P-value**	**95% Confidence Interval**	
Medicare Advantage (Reference= privately insured)	-8970.24	0.00	-9877.13	-8063.35
Total drug units	4.01	0.00	2.07	5.94
**Drug (Reference=Bevacizumab)**				
Filgrastim	-51756.17	0.00	-53956.31	-49556.04
Pegfilgrastim	-29646.55	0.00	-31857.02	-27436.07
Rituximab	-26068.52	0.00	-28248.61	-23888.44
Trastuzumab	27773.66	0.00	23957.78	31589.54
**Year (Reference=2007)**				
2008	895.60	0.70	-3634.38	5425.57
2009	-1245.93	0.58	-5625.91	3134.05
2010	701.01	0.75	-3563.25	4965.27
2011	3771.55	0.09	-591.11	8134.21
2012	3150.70	0.16	-1217.33	7518.73
2013	4479.44	0.05	21.38	8937.49
2014	4754.89	0.05	7.23	9502.55
2015	6795.26	0.01	1973.07	11617.45
2016	8609.89	0.00	3687.63	13532.15
2017	11265.99	0.00	6201.88	16330.10
2018	16434.33	0.00	11242.64	21626.03
2019	11996.51	0.00	6725.30	17267.72
Diff-in-diff **(Reference t=0)**				
t=-3	180.73	0.90	-2651.29	3012.74
t=-2	-760.73	0.56	-3343.93	1822.46
t=-1	1539.38	0.24	-1021.74	4100.50
t=1	4074.77	0.00	1592.84	6556.70
t=2	2722.34	0.02	364.68	5080.00
t=3	2521.17	0.05	36.03	5006.31

Note: Regression model also included the following variables not reported in the table above: indicators for hospitals, event time, sex, state, age categories and interaction of age categories and sex are suppressed from output.

© 2023 Chang J et al. *JAMA Health Forum.*

eTable 2. Characteristics of patient episodes

Variable	340B		Non-340B	
	N	%	N	%
**Drug**				
Bevacizumab	4,194	11.67%	7,431	11.92%
Filgrastim	6,304	17.55%	11,252	18.05%
Pegfilgrastim	14,478	40.30%	25,566	41.00%
Rituximab	8,728	24.29%	14,108	22.63%
Trastuzumab	2,225	6.19%	3,997	6.41%
**Insurance**				
Commercial	23,209	66.65%	47,493	78.75%
Medicare Advantage	11,612	33.35%	12,813	21.25%
**Age Group**				
< 18	900	2.58%	511	0.85%
18-44	3,636	10.44%	8,128	13.48%
45-64	14,108	40.52%	28,282	46.9%
65+	16,177	46.46%	23,385	38.78%
**Sex**				
Female	20,937	60.13%	35,980	59.66%
Male	13,873	39.84%	24,313	40.32%
Unknown	11	0.03%	13	0.02%
**N**	34,821		60,306	

**where AHA data elements were available at t=0 (year of 340B program participation)*

*** Percent displayed in adherence to data vendor's cell size suppression policy*

Robustness checks

Callaway and Sant'anna propose an alternative estimator of the main event study analysis of treatment effects over time that is complicated by the fact that different event times (i.e., times relative to the intervention date) have different compositions of groups because of staggered participation dates. They propose a reaggregation that averages effects by length of exposure. **EFigure1** presents this alternative estimator. Patterns are similar to the main event study analysis presented in the text. See the Callaway Sant'anna paper for further detail: Callaway B, Sant'Anna PHC. Difference-in-Differences with multiple time periods. Journal of Econometrics. 2021;225(2):200-230.

© 2023 Chang J et al. *JAMA Health Forum.*

eFigure 1. Callaway-Sant'Anna average effect by length of exposure

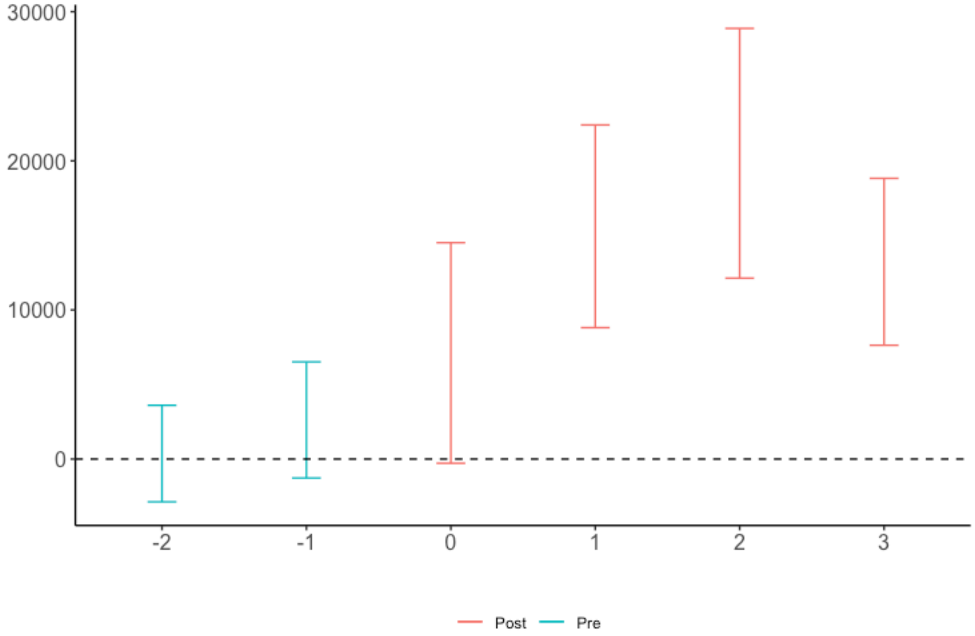

Note: Error bars indicate 95% confidence intervals.

Callaway and Sant'anna also suggest analyzing heterogeneity of group-time effects to assess possible cohort effects and the generalizability of the overall estimated effect to the different cohorts. EFigure2 presents average effects across the post-participation period for each participation cohort. We see considerable heterogeneity in group-time effects across the cohorts. The hospitals initiating 340B participation in 2012, 2013, and 2015 had large, positive treatment effects. The cohorts initiating in 2010, 2011, 2014, and 2016 had treatment effects not statistically distinguishable from zero. No cohorts showed negative treatment effects. ETable 3 presents volume comparisons across the cohorts.

While we were unable to identify the specific qualification of 340B program participation in our data, we hypothesize that the early 340B program participants were smaller, and therefore had less market power, and may have automatically qualified for the 340B program with the passage of the Affordable Care Act in 2010. We tested this hypothesis by dichotomizing our 340B (treated) hospitals as "early treatment" if they started participating in 340B program in 2010 and 2011. We found that early treated hospitals were more likely to be smaller (22.09% of early 340B hospitals had a bed size code of 1 to 49 beds compared to 10.26% of 340B hospitals participating after 2011). In addition, we observed lower average inpatient admissions between early and later 340B-participating hospitals (22,031.70 vs. 31,131.09; p-value=0.0210) and lower average Medicare discharges between early and later 340B-participating hospitals (3031.11 vs. 6419.46; p-value=0.1267).

© 2023 Chang J et al. *JAMA Health Forum.*

eFigure 2. Callaway-Sant'Anna group-time effects

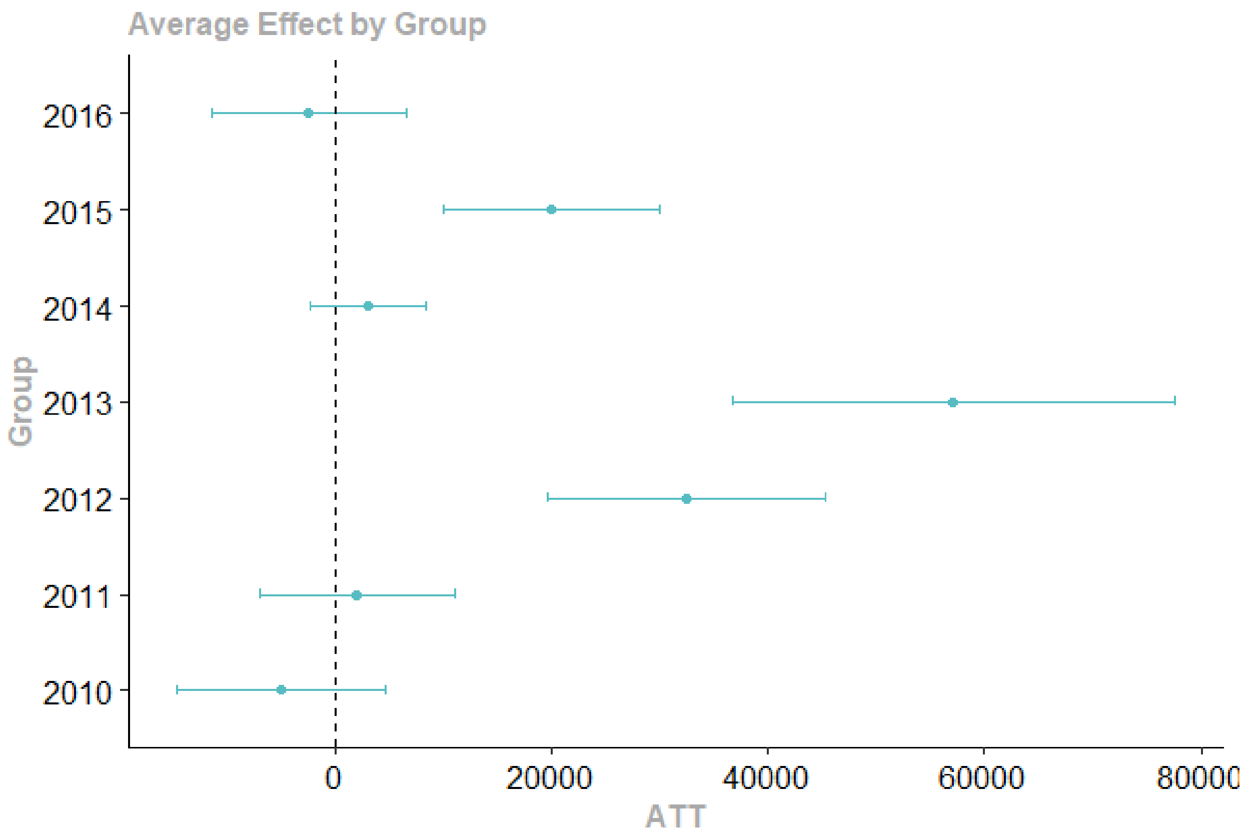

Note: Error bars indicate 95% confidence intervals.

© 2023 Chang J et al. *JAMA Health Forum.*

eTable 3. Additional descriptive analysis of hospital sample

340B participation year	Average admissions*	Total outpatient visits*
2010		
Control	25,418	266,669
Treated	22,062	300,742
2011		
Control	27,573	252,345
Treated	21,978	221,495
2012		
Control	30,003	436,030
Treated	44,596	382,906
2013		
Control	28,066	271,362
Treated	25,016	304,053
2014		
Control	30,640	398,244
Treated	18,045	228,106
2015		
Control	27,291	358,592
Treated	31,072	348,013
2016		
Control	32,760	411,894
Treated	33,899	357,931

*Data derived from American Hospital Association (AHA) Annual Survey

© 2023 Chang J et al. *JAMA Health Forum.*

eMethods

Analytical episode data identification

The main unit of analysis is patient-drug episode treatment, which comprises of all outpatient claims, defined as drugs administered in hospital outpatient department, during the episode. Hospital outpatient claims are identified using the facility UB-04 claim form. Excluded from this analysis are administered drug claims from office settings using the physician CMS-1500 claim form, i.e., non-hospital physician offices. This study excluded drug episodes in office setting because of data limitations from the data source to identify hospital affiliation. Episodes with evidence of drug administrations in multiple hospitals were also excluded from the study sample. We also excluded episodes where health plan was *not* the primary payer e.g. worker's compensation or supplemental health plans. Hospitals were identified as 340B program participants using hospital participation data from the Health Resources and Services Administration.

Our primary outcome of interest was the total allowed amount for each drug treatment episode, including health insurance plan-paid and patient-paid amount. The total allowed amount only included administered drugs (identified through HCPCS codes); we excluded other services such as administrative fees, labs, and radiological exams. While unit price analysis, (total allowed amounts per billed unit) is the preferred price outcome, various payment arrangements between health plans and providers make it difficult to calculate the unit price meaningfully and correctly for each drug from the facility claims. For that reason, we sum all episode costs for each patient-treatment episode.

Primary regression specification

$$p_{i,h,d,t} = \beta_0 + \beta_2 Treat_{ht} A_{h,t} + \eta_t + \gamma_h + \delta_d + \alpha_i + \varepsilon_{h,i,d,t}$$

where

- i denotes patient, h denotes hospital, t denotes calendar year and event time, and d denotes drug.
- η_t denotes vector calendar year and event time indicators
- γ_h denotes vector of de-identified hospital indicators
- δ_d denotes vector of drug indicators
- α_i denotes vector of patient characteristics such as insurance segment, age band, state of residence, and sex
- $Treat_{ht} A_{h,t}$ denotes interaction of event-time indicators and 340B program participation indicator

© 2023 Chang J et al. *JAMA Health Forum.*

Additional results of main specification by type of drug

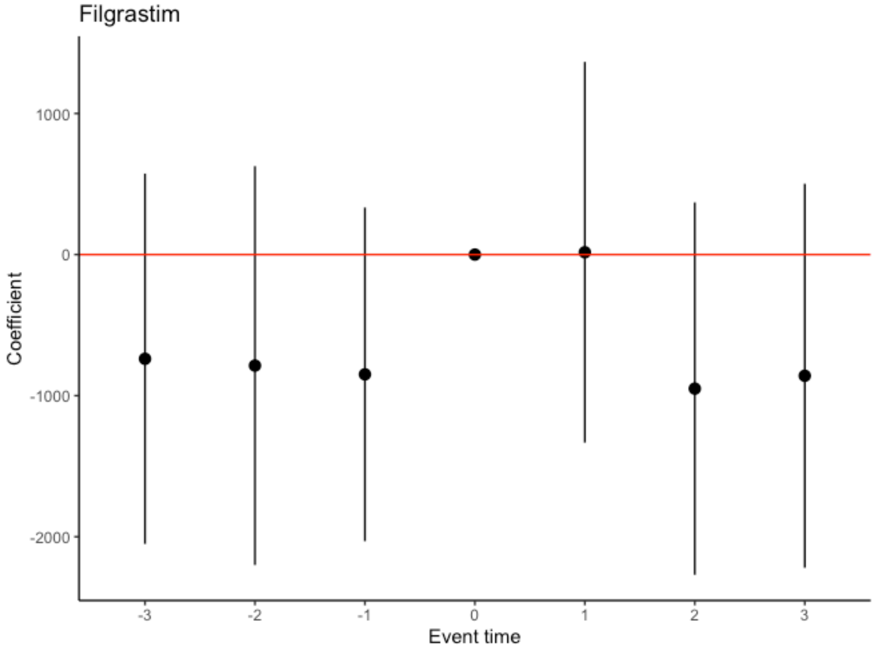

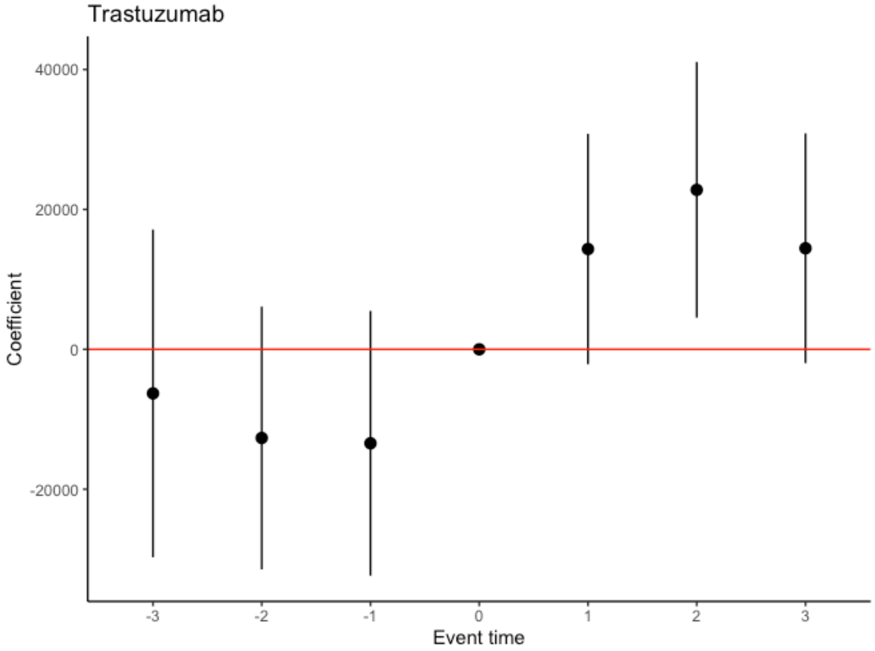

© 2023 Chang J et al. *JAMA Health Forum.*

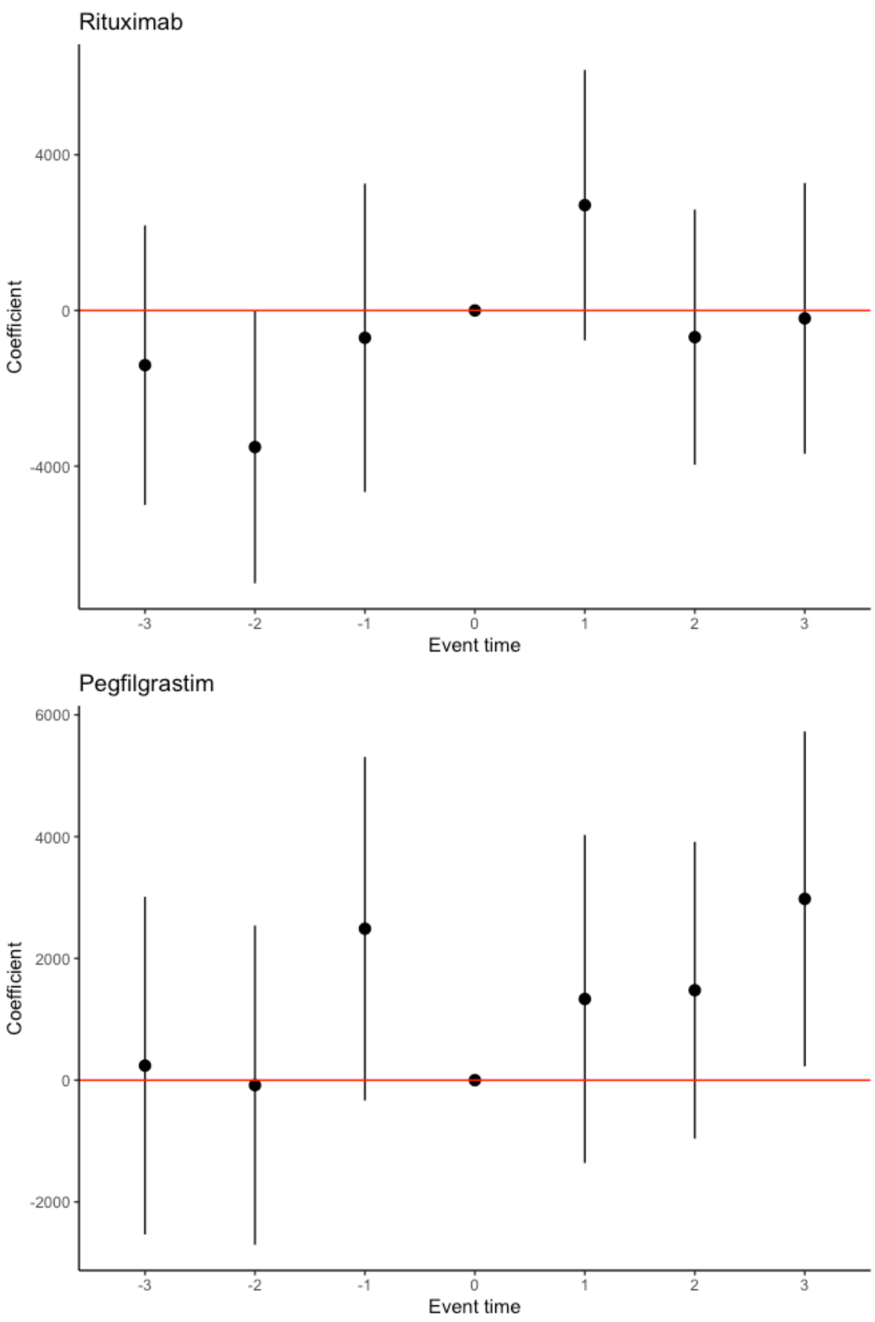

© 2023 Chang J et al. *JAMA Health Forum.*

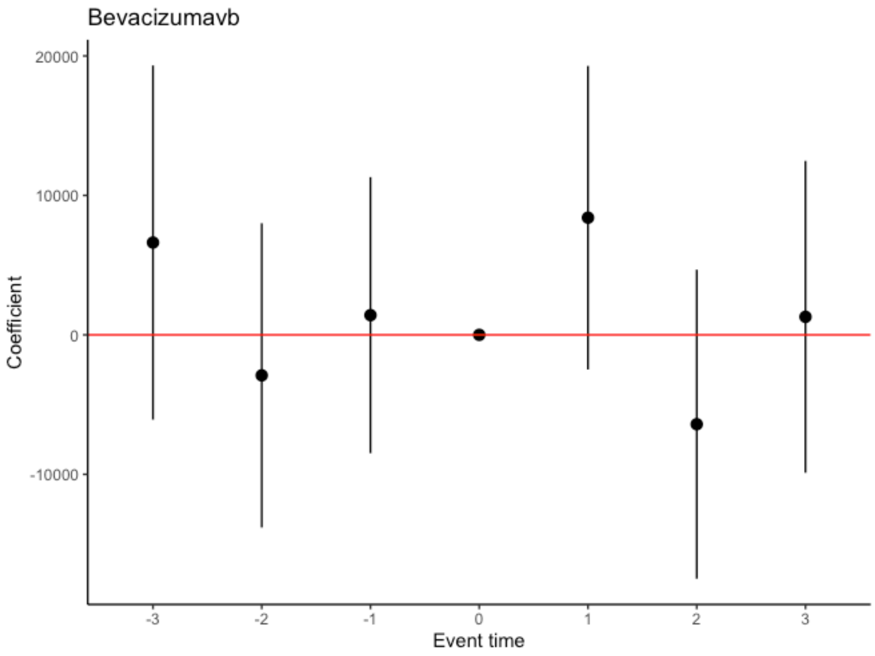

© 2023 Chang J et al. *JAMA Health Forum.*
