# Supplementary material for: Association Between New 340B Program Participation and Commercial Insurance Spending on Outpatient Biologic Oncology Drugs
Source: JAMA Health Forum. 2023 Jun 23;4(6):e231485. doi: 10.1001/jamahealthforum.2023.1485 (PMC10290244; doi:10.1001/jamahealthforum.2023.1485)
Supplement: Supplement 2. — Data Sharing Statement [file jamahealthforum-e231485-s002.pdf]

Data Sharing Statement

Chang. Association Between New 340B Program Participation and Commercial Insurance Spending on Outpatient Biologic Oncology Drugs. *JAMA Health Forum*. Published June 23, 2023. doi:10.1001/jamahealthforum.2023.1485

Data
Data available: No

Additional Information
Explanation for why data not available: The data use agreement with the data provider does not allow us to share patient-level data
